# Integrated oral microbiome and metabolome analysis unveils key biomarkers and functional pathway alterations in patients with acute myocardial infarction

**DOI:** 10.3389/fcimb.2025.1607845

**Published:** 2025-07-31

**Authors:** Ikram Khan, Muhammad Irfan, Imran Khan, Xie Ping, Yu Xiaohui, Shengnan Lei, Tianzhu Song, Xiaodong Xie, Zhiqiang Li

**Affiliations:** ^1^ Department of Genetics, School of Basic Medical Sciences, Lanzhou University, Lanzhou, Gansu, China; ^2^ Department of Medical Laboratory Technology, Xcito School of Nursing and Allied Health Sciences, Chakdara, Khyber Pakhtunkhwa, Pakistan; ^3^ Department of Microecology, School of Basic Medical Sciences, Dalian Medical University, Dalian, Liaoning, China; ^4^ Department of Cardiology, Gansu Province People’s Hospital, Lanzhou, Gansu, China; ^5^ Department of Gastroenterology, The 940 Hospital Joint Logistic Support Force of People's Liberation Army (PLA), Lanzhou, Gansu, China; ^6^ School of Stomatology, Key Laboratory of Oral Disease, Northwest Minzu University, Lanzhou, Gansu, China

**Keywords:** microbial dysbiosis, metabolite dysregulations, pathway disruptions, noninvasive biomarkers, AMI

## Abstract

**Introduction:**

The significance of oral microbiota in acute myocardial infarction (AMI) has been increasingly appreciated. However, the association between oral microbiota, metabolites, and AMI parameters, as well as the key microbiota and metabolites that may play a crucial role in this process, remains unclear. To investigate the oral microbiome composition and metabolomic profiles associated with AMI and explore the roles of specific bacterial species and key metabolites in functional pathways in the progression of AMI.

**Methods:**

A case-control study was conducted involving 24 AMI patients and 24 matched healthy controls. Saliva samples were collected for 16S rRNA sequencing and untargeted LC-MS metabolomics. Correlation analysis was then performed to explore associations between microbial taxa, metabolomic profiles, and AMI clinical parameters, with results visualized as heatmaps of correlation coefficients.

**Results:**

The component of the oral microbiota of the AMI group showed significant alterations when compared to the control group. Particularly, a significant shift in terms of alpha and beta diversity was observed between the AMI and control groups (p < 0.05). The *Streptococcus* and *Rothia* genera, as well as 9(R)-HODE, 20-HETE ethanolamide, and 5,6 EET metabolites, were identified as potential biomarkers, achieving an area under the curve of 0.82–0.88. Functional pathway analysis demonstrated significant upregulation in key metabolic pathways, including the Citrate Cycle (TCA cycle), Pyruvate metabolism, and Glucagon signaling pathways, which exhibited strong correlations with established clinical markers of AMI.

**Conclusion:**

This integrative analysis underscores the diagnostic potential of oral microbiome-metabolome interactions in AMI and unveils key mechanistic pathways for guiding future therapeutic interventions.

## Introduction

Acute myocardial infarction is a common and critical cardiovascular event associated with significant morbidity and mortality (Ferrero, 2023). As a major global contributor to heart failure and cardiac-related deaths, AMI constitutes a substantial burden on public health systems worldwide (Alnemer, 2024). While percutaneous coronary intervention (PCI) has significantly reduced mortality associated with AMI, there remains a critical need for advanced strategies to enhance therapeutic efficacy and long-term patient outcomes (Buonpane et al., 2024). Recent studies suggest that the gut microbiome is a dynamic and adaptable environmental factor that may influence the pathogenesis of AMI and holds promise as a potential therapeutic target (Rahman et al., 2022). However, despite being the second-largest microbial community (Li et al., 2024), the role of oral microbiota in the pathogenesis of AMI remains poorly understood.

Growing evidence indicates the association between oral microbiota and AMI, with studies showing that serum antibody levels against various oral pathogenic microorganisms are positively associated with an increased risk of myocardial infarction (MI) (Joshi et al., 2021). Polymerase chain reaction (PCR) and microbial sequencing technologies have identified oral microbial DNA, including periodontal and non-periodontal pathogenic bacteria, in coronary artery thrombi of MI patients (Kwun et al., 2020), suggesting a potential link between oral bacteria and the formation of coronary artery thrombi in AMI. Periodontitis induced by the disruption of oral microbial ecology has been documented to raise the risk of AMI (Carra et al., 2023; Bijla et al., 2024). However, the specific alterations in the oral microbiome and metabolomic profile associated with AMI remain insufficiently characterized.

Integrative analysis of microbiome and metabolomics has become a powerful tool for uncovering the complex interactions between microbiome dynamics and health outcomes (Feng et al., 2019; Zhao et al., 2021). Metabolites, as small molecules that reflect underlying biological processes, are widely used in clinical medicine as biomarkers for diagnosis, prognosis, and assessment of treatment efficacy (Schmidt et al., 2021). Advances in high-throughput technologies now allow systematic profiling of the metabolome, offering comprehensive insights into cardiometabolic alterations. Blood-based metabolomic studies have identified disease-associated pathways, notably those linked to amino acid and fatty acid metabolism (Fan and Pedersen, 2021). In addition, cutting-edge metabolomics studies have provided valuable insights into the risk of type 2 diabetes (T2D) and its complications, with analyses conducted on diverse biological samples, including plasma, serum, and urine (Morze et al., 2022). A previous study explored the association between paired plasma and salivary metabolomic datasets in patients with T2D, highlighting the potential utility of salivary metabolites as biomarkers for assessing systemic metabolic dysfunction (Sakanaka et al., 2021). These findings emphasize the oral microbiome’s potential as a powerful tool for disease discrimination and early diagnosis. Expanding on this evidence, investigating the roles of the oral microbiome and metabolome in cardiovascular diseases (CVDs), especially AMI, is essential given its significant global health burden.

Therefore, this study investigated the interplay between the oral microbiome and metabolomic profiles in AMI patients and healthy controls, aiming to identify potential biomarkers and underlying pathways associated with CVDs, particularly AMI. Integrated analysis of the microbiome and metabolome revealed significant alterations in the oral microbiome composition, metabolomic profile, and functional pathways in AMI patients. We further explored the interaction between the microbiome, metabolome, and clinical markers to gain a deeper understanding of their interrelationships. Understanding these interactions could inform the development of novel diagnostic biomarkers or therapeutic targets for AMI. These findings provide a foundation for leveraging clinical metagenomics to identify novel biomarkers and understand the mechanistic pathways underlying AMI.

## Materials and methods

### Study cohort and patient characteristics

Samples were collected from a total of 48 individuals, consisting of 24 participants who had been diagnosed with acute myocardial infarction (AMI) and 24 controls. The participants were recruited from the Department of Cardiology, Gansu Provincial People’s Hospital, and the Department of Physical Examination, the 940 Hospital Joint Logistic Support Force of PLA. Diagnosis of AMI was performed based on established guidelines (Members et al., 2012). All participants were between the ages of 30 and 60. Exclusion criteria included ongoing infectious diseases, cancer, renal or hepatic failure, peripheral neuropathy, stroke, and recent antibiotic use within 3 months. The control group was comprised of individuals who did not exhibit any clinically evident symptoms of AMI when recruited. Clinical and demographic data were collected for all participants, and individuals with incomplete data were excluded at the time of recruitment.

### Oral examination of the participants

All study participants underwent a detailed oral examination conducted by a single, calibrated dentist to assess variables potentially influencing the oral microbiome. The examination revealed no clinical manifestations of periodontal disease, including gingivitis or periodontitis. The prevalence of dental caries was negligible, with teeth generally observed to be in excellent condition, exhibiting minimal wear, no visible decay, and an absence of previous restorations. Gingival tissues appeared healthy, characterized by a pink coloration, firm texture, and tight adherence to the teeth, with no evidence of bleeding, inflammation, or other pathological signs. Furthermore, no active oral lesions were identified, such as ulcers, sores, or neoplastic changes indicative of oral malignancies. Participants demonstrated adequate oral hygiene, maintaining a normal complement of functional teeth that required only routine care. Notably, none of the participants had undergone professional dental cleaning within the preceding year or received periodontal treatment in the 3 months before enrollment, ensuring standardized and comparable oral health conditions across the cohort.

### Sample collection

Samples were collected from each patient on the first day of hospital admission, before primary coronary interventions. All participants were instructed to gargle and avoid eating and drinking for at least 1 hour before oral sample collection. Saliva samples were collected in 50 ml centrifuge tubes with saliva DNA preservation solution. All samples were transported to the laboratory on ice packs within 2 hours after collection and stored at -80°C.

Blood samples were collected after an overnight fast of at least 8 hours for clinical chemistry analyses. The samples were collected in tubes with anticoagulants, centrifuged at 3500 rpm for 15 min, and the supernatants were stored at 80°C. Blood variables, such as systolic blood pressure and diastolic blood pressure, were measured in mmHg values. Serum biomarkers, including Low-density lipoprotein, High-density lipoprotein, Total cholesterol, Triglycerides, Blood glucose, C-reactive protein, Uric acid, White and Red blood cell counts, Platelet count, Hemoglobin, Neutrophil count, Serum creatinine, and pH, were assessed during the initial clinical screening. In addition, Age was measured in years, body mass index (BMI) was calculated as weight in kilograms divided by height in meters squared (kg/m²), and blood pressure was measured in mmHg for both systolic and diastolic values.

### DNA extraction and amplicon sequencing

DNA was extracted from 100 µl of saliva with the TGuide S96 Magnetic Soil/Stool DNA Kit, Biomarker Technologies Co., Ltd., Beijing, China, following the manufacturer’s instructions. The DNA concentration of the samples was measured with the Qubit dsDNA HS Assay Kit and Qubit 4.0 Fluorometer (Invitrogen, Thermo Fisher Scientific, Oregon, USA). The extracted DNA was stored at –80 °C for subsequent analyses (Khan et al., 2022).

The 338F: 5’- ACTCCTACGGGAGGCAGCA-3’ and 806R: 5’- GGACTACHVGGGTWTCTAAT-3’ universal primer set was used to amplify the V3-V4 region of the 16S rRNA gene from the genomic DNA extracted from each sample (Khan et al., 2022). Sample-specific Illumina index sequences were appended to both forward and reverse 16S primers for high-throughput sequencing. PCR was carried out in a 10 μl reaction containing 5–50 ng DNA template, 0.3 μl each of \*Vn Forward and \*Vn Reverse primers (10 μM), 5 μl KOD FX Neo Buffer, 2 μl dNTPs (2 mM each), 0.2 μl KOD FX Neo polymerase, and nuclease-free water to volume. The Vn forward and reverse primers were selected based on the targeted amplification region. Thermal cycling conditions included an initial denaturation at 95°C for 5 min, followed by 25 cycles of 95°C for 30 seconds, 50°C for 30  seconds, and 72°C for 40  seconds, with a final extension at 72°C for 7 minutes. All the PCR amplicons were purified using Agencourt AMPure XP beads (Beckman Coulter, Indianapolis, IN) and quantified with the Qubit dsDNA HS Assay Kit on a Qubit 4.0 Fluorometer (Invitrogen, Thermo Fisher Scientific, Oregon, USA). Following individual quantification, amplicons were pooled in equimolar amounts and sequenced on the Illumina Novaseq 6000 platform (Illumina, San Diego, CA, USA).

### Bioinformatic analysis

The bioinformatics analysis of this study was performed with the aid of the BMK Cloud (Biomarker Technologies Co., Ltd., Beijing, China). Raw reads were quality-filtered using Trimmomatic (v0.33) (Bolger et al., 2014), with primer removal performed by Cutadapt (v1.9.1) (Martin, 2011). FLASH (v1.2.11) was used to trim terminal reads (Magoč and Salzberg, 2011), UCHIME (v8.1) to remove chimeras (Edgar et al., 2011), and USEARCH (v10.0) to discard sequences shorter than 100 bp or exceeding a 2% error rate (Edgar, 2013). Sequences were taxonomically classified using the Ribosomal Database Project http://www.arb-saliva.de. High-quality reads were clustered into operational taxonomic units (OTUs) at a 97% similarity threshold (Bokulich et al., 2013). Rarefaction curves at the OTUs level and Venn diagrams were generated in R (v4.2.1). Alpha diversity metrics, Chao1 index (Chao, 1984), and Shannon index (Shannon, 1948), were analyzed using Mothur software (V1.30) http://www.mothur.org/, and Beta diversity analysis to compare the similarity of species diversity between different samples. Beta diversity assessed via Principal Coordinate analysis (PCoA) based on Bray–Curtis dissimilarity metrics (Lozupone et al., 2007). Group differences in relative abundance were tested using the Wilcoxon rank-sum test. Linear discriminant analysis Effect Size (LefSe) was applied to identify discriminatory taxa, with Linear discriminant analysis (LDA) scores supported by pairwise Wilcoxon tests (Bokulich et al., 2013). Functional prediction of oral microbiota was performed using PICRUSt2 based on Clusters of Orthologous Groups (COG) annotations.

### Metabolite extraction and LC-MS analysis

The LC/MS system used for metabolomics analysis includes a Waters Acquity I-Class PLUS UHPLC system and a Waters Xevo G2-XS QT of high-resolution mass spectrometer, equipped with a Waters Acquity UPLC HSS T3 column (1.8 µm, 2.1 x 100 mm). For both positive and negative ion modes, the mobile phases are 0.1% formic acid in water (A) and 0.1% formic acid in acetonitrile (B), with an injection volume of 1 µL. The Waters Xevo G2-XS QT high-resolution mass spectrometer, controlled by MassLynx (V4.2) software, operates in MSe mode to acquire primary and secondary mass spectrometry data. Each acquisition cycle captures dual-channel data at low 2 volts and elevated 10–40 volts collision energies with a scanning frequency of 0.2 seconds per spectrum. The ESI ion source parameters are as follows: capillary voltage is set to 2000 volts for positive ion mode and -1500 volts for negative ion mode, with a cone voltage of 30 volts. The ion source temperature is maintained at 150°C, while the desolvation gas temperature is 500°C. The backflush gas flow rate is 50 L/h, and the desolvation gas flow rate is 800 L/h.

Raw data acquired from MassLynx is processed through Progenesis QI software, which performs peak extraction, alignment, and various analytical tasks. Compound identification is conducted using the online METLIN database and Biomark’s custom-built library within Progenesis QI software. Theoretical fragment identification is carried out with mass deviations controlled within 100 ppm. Following normalization of the peak area data by the total peak area, subsequent analyses were performed. Principal Component Analysis (PCA) and Spearman correlation analysis were employed to assess the repeatability of samples within groups and the quality control samples. Using the R package ropls, Orthogonal partial least squares discriminant analysis (OPLS-DA) modeling was performed, incorporating 200 permutation tests to validate model reliability. The Variable Importance in Projection (VIP) value of the model was determined through extensive cross-validation. Differential metabolites were identified according to Fold Change (FC > 1), p-value (*p* < 0.01), and VIP value (VIP > 1). The Receiver Operating Curve (ROC) analysis was performed to assess key metabolites’ diagnostic ability for AMI diagnosis using the R package pROC. Fold changes (FC) were calculated and compared based on group classifications, with t-tests determining the statistical significance of differences for each compound. The Kyoto Encyclopedia of Genes and Genomes (KEGG) functional prediction pathways of the salivary metabolomics were inferred using Phylogenetic Investigation of Communities by Reconstruction of Unobserved States (PICRUSt2). To reduce false positives, p-values were adjusted using the Benjamini-Hochberg method for multiple testing, applying a significance cutoff of False Discovery Rate < 0.05.

### Statistical analysis

Descriptive statistics were used to assess baseline characteristics; quantitative variables are presented as mean and standard deviation (±), and categorical variables as percentages. Group differences in clinical indices were assessed using the Student’s *t*-test for normally distributed quantitative variables or the nonparametric Mann–Whitney test for non-normally distributed variables. Spearman’s correlation coefficient was used to assess relationships of oral microbiota with salivary metabolites and clinical parameters, visualized via the R package “pheatmap.” Statistical significance was defined as a p-value of ≤ 0.05.

## Results

### Baseline characteristics of the participants

We enrolled forty-eight participants in this study, comprising twenty-four patients with AMI and twenty-four healthy controls. Detailed demographic and clinical characteristics are presented in our previous study (Khan et al., 2025). No statistically significant differences were observed in sex, smoking status, diabetes, diastolic blood pressure, total cholesterol, uric acid, platelets, and serum creatinine between the AMI and control groups. However, AMI patients exhibited significantly higher levels of triglycerides, Low-density lipoprotein cholesterol, and C-reactive protein, along with lower high-density lipoprotein cholesterol levels (*p* < 0.05), suggesting a potential role for these covariates in AMI progression and their possible association with oral microbiome composition. AMI patients were older than controls, likely contributing to the observed differences. Future studies should stratify by age in multivariate models to address its potential confounding effects on oral microbial composition and metabolomic profile.

### OTUs distribution and microbial diversity indexes

**Figure 1:**
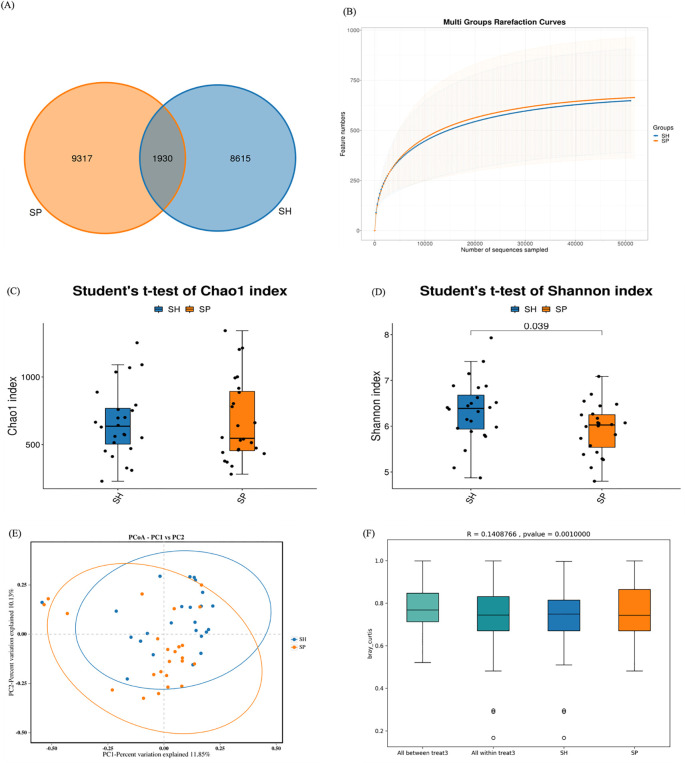
OTUs distributions and diversity indexes between the two groups. **(A)** The Venn diagram shows the observed OTUs between healthy and AMI groups, with overlapping OTUs denoted by the intersection area among corresponding circles. **(B)** Rarefaction curves depict microbiota diversity from saliva samples. **(C)** The Chao1 index between the AMI and control group. **(D)** Shannon index between the AMI and control groups. **(E)** Principal coordinate analysis displays the distance-based similarity/dissimilarity of saliva samples between the AMI and Control groups. **(F)** PERMANOVA analysis confirms the significant differences in beta diversity between the AMI and control groups. SH represents the healthy group, and SP represents the AMI group.

Sequencing of 48 saliva samples yielded 3,798,626 raw reads. After quality enhancement through paired-end read splicing, 3,471,942 clean reads were obtained, averaging 72,332 clean reads per sample. A total of 19,862 OTUs were obtained from both groups. Of them, 8,615 OTUs were unique to the control and 9,317 to the AMI group, while 1,930 OTUs were common in both groups (**Figure 1A**). A rarefaction curve suggested that sequencing depth was adequate (**Figure 1B**). Alpha diversity analysis was conducted to assess the microbial composition, diversity, and evenness within the groups. No significant difference was observed in the Chao1 index between patients and controls (*p* = 0.88; **Figure 1C**), whereas the Shannon index differed significantly between AMI and controls (*p* = 0.03; **Figure 1D**). Beta diversity analysis was then conducted to assess differences in microbial community composition between the two groups. The separation shown in the Bray-Curtis principal coordinate analysis diagram was apparent (**Figure 1E**), and PERMANOVA analysis (R^2^ = 0.140; *p* = 0.001) validated the significant differences between both groups (**Figure 1F**). Overall, alpha and beta diversity analyses indicated substantial differences between AMI and control groups, suggesting a potential role of microbial dysbiosis in AMI.

### Relative abundance of oral microbiota between both groups

**Figure 2:**
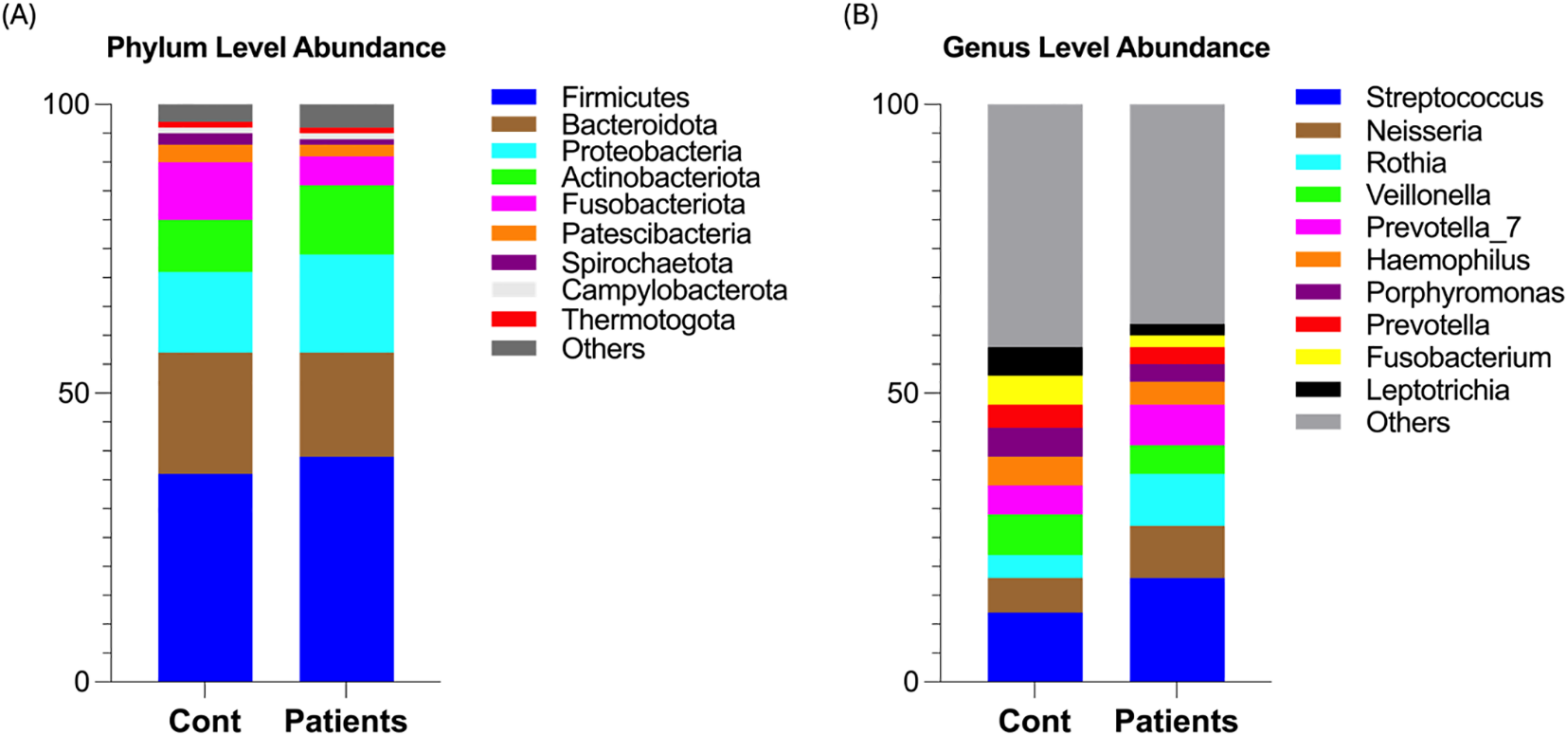
Relative abundance of oral microbiota between both groups. **(A)** Shows phylum-level microbial abundance in AMI patients and healthy controls. **(B)** Shows genus-level abundance in both groups.

We next characterized the microbial abundance between healthy and patient groups. Firmicutes was the most abundant phylum (39.04% in AMI vs. 36.44% in controls), followed by Proteobacteria (17.86% vs. 14.68%) and Actinobacteriota (12.03% vs. 9.06%), while Bacteroidota (18.85% vs. 21.10%) and Fusobacteriota (5% vs. 10%) were reduced in AMI patients (**Figure 2A**). At the genus level, *Streptococcus* exhibited the highest prevalence, with a relative abundance of (18.67% vs. 12.43%), followed by *Neisseria* (9.10% vs. 6.66%), *Rothia* (8.97% vs. 4.54%), and *Prevotella_7* (7.40% vs. 5.79%), with depleted levels of *Veillonella_7* observed in the salivary microbiota of the MI group compared to the control group (**Figure 2B**). These findings suggest that AMI patients exhibit a dysbiotic oral microbiota, marked by an enrichment of Firmicutes (e.g., *Streptococcus*), Proteobacteria, and *Rothia*, alongside a depletion of Bacteroidota, Fusobacteriota, and *Veillonella_7*. This microbial dysbiosis may contribute to the pathophysiological mechanisms underlying myocardial infarction.

### Oral microbiota-based biomarkers for AMI

**Figure 3:**
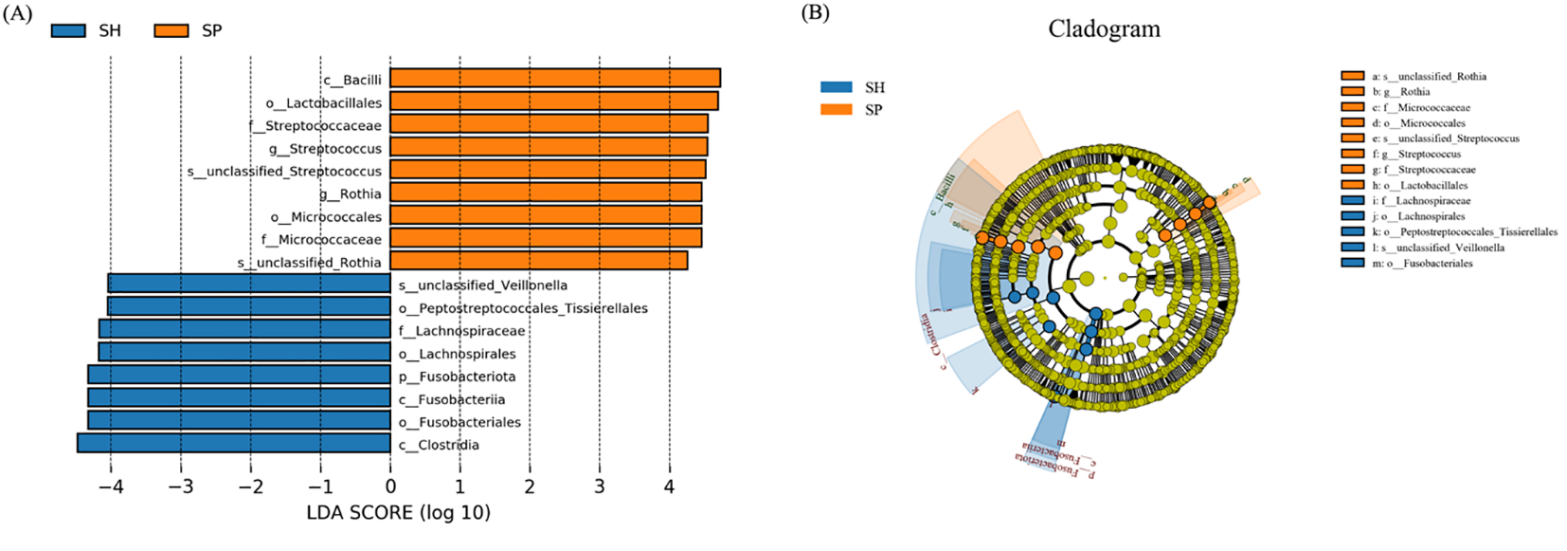
Distinct microbial taxa between the AMI and control groups. **(A)** Using an LDA score threshold of 3, LEfSe identified the most significantly differentially abundant clades across various taxonomic levels between the AMI and control groups. **(B)** The cladogram illustrates the distribution of these differentially abundant bacterial taxa, with each layer representing a distinct taxonomic level. SH represents the healthy group, and SP represents the AMI group.

LEfSe analysis was performed to further investigate oral microbiome differences between AMI patients and controls. Seventeen distinct bacterial taxa were identified between groups, including Bacilli, Lactobacillales, Micrococcales, Streptococcacae, Micrococcaceae, *Streptococcus*, and genus *Rothia* were detected in the AMI group, and Fusobacteriota, Fusobacteria, Clostridia, Peptostreptococcales_Tissierellales, and Fusobacteriales, Lachnospiraceae were distinct in control groups (**Figure 3A**). The cladogram depicts taxa with significant differences between control and AMI groups, organized hierarchically from phylum to genus (**Figure 3B**). These findings underscore the influence of AMI on the stability and composition of the oral microbiome, potentially contributing to the underlying pathophysiological mechanisms of the disease.

### Predicted functional pathways of oral microbiota in AMI

**Figure 4:**
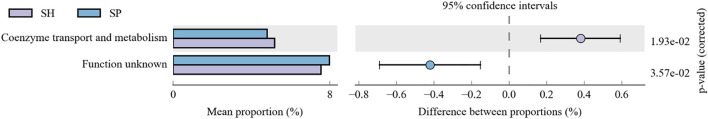
Functional prediction pathways analysis between both groups. The functional pathways analysis of oral microbiota reveals significant dysregulation in two pathways between the AMI and control groups. SH represents the healthy group, and SP represents the AMI group.

Next, COG annotation was used via PICRUSt2 to analyze the biological and functional profiles of the oral microbiota. Our findings revealed that two functional pathways were significantly dysregulated between AMI and control groups, including Coenzyme transport and metabolism were downregulated considerably in AMI patients, and the function-unknown pathway was significantly upregulated in AMI patients than the control group (**Figure 4**). These findings suggest that AMI is associated with functional alterations in the oral microbiota, including impaired coenzyme metabolism, potentially affecting microbial energy processes, and the activation of uncharacterized pathways, highlighting novel microbial involvement in AMI.

### Metabolomic profiling between both groups

**Figure 5:**
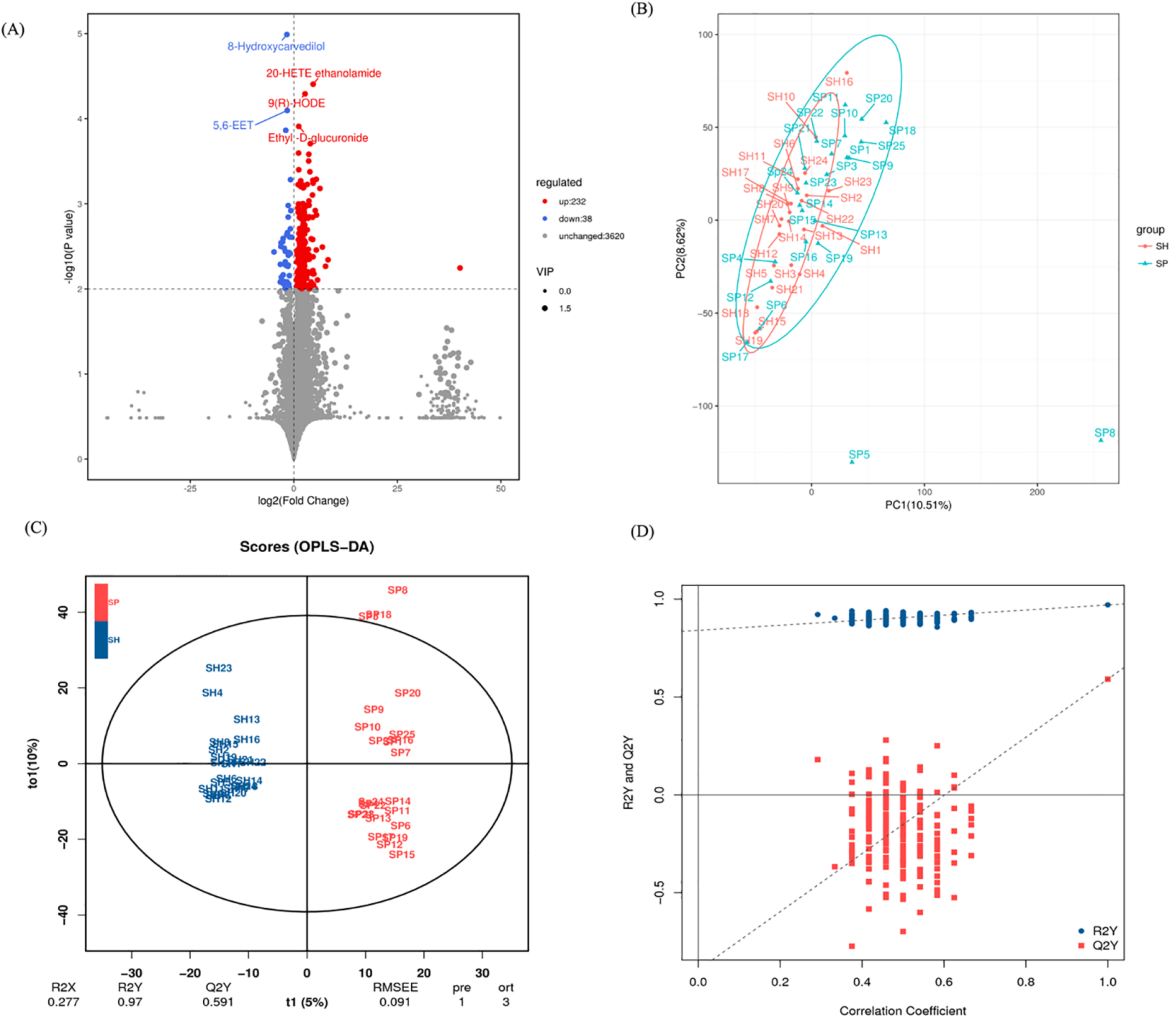
Salivary metabolites signature between the AMI and control group. **(A)** The volcano plot identifies metabolites that differ between the AMI and control groups. **(B)** PCA shows distinct metabolite profiles for both groups. **(C)** OPLS-DA emphasizes metabolite differences between the AMI and control groups. **(D)** A permutation test confirms the validity of the OPLS-DA model, distinguishing AMI and control groups. SH represents the healthy group, and SP represents the AMI group.

We subsequently performed untargeted metabolomic analysis on saliva samples to investigate differences in metabolite profiles between AMI patients and control groups. The volcano plot provides a visual representation of the metabolomic differences between the AMI and control groups. A total of 270 metabolites were identified across both groups, with 232 metabolites upregulated and 38 downregulated between the AMI and control groups (**Figure 5A**). Five metabolites, including 8-Hydroxycavedilol and 5,6 EET metabolites, were significantly downregulated, and 20-HETE ethanolamide, 9(R)-HODE, and Ethyl-D-glucuronide were upregulated in the AMI group.

Principal component analysis (PCA) showed metabolic differences between the groups, with PC1 and PC2 explaining 10.51% and 8.62% of the variation, respectively (**Figure 5B**). Orthogonal partial least squares to latent structure discriminant analysis (OPLS-DA) models indicated that the metabolic composition of AMI patients was differentiated from the control group (**Figure 5C**). Model robustness was confirmed through cross-validation and permutation tests, ensuring the reliability of metabolite differentiation between AMI and control groups (**Figure 5D**). These changes indicate significant metabolic disruptions in AMI patients, likely driven by oral microbiota and host-microbe co-metabolism. The distinct metabolomic profiles suggest potential biomarkers and pathways linked to AMI pathophysiology.

### Salivary metabolite-based biomarkers

**Figure 6:**
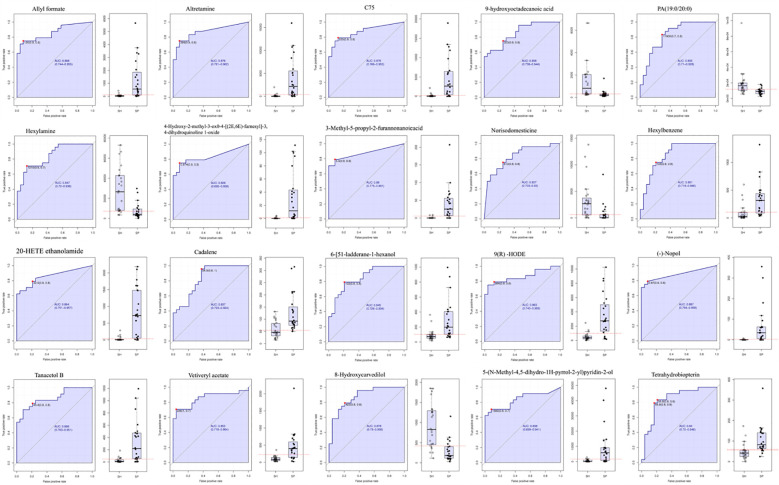
ROC analysis for discrimination of AMI patients from the control group. Receiver Operating Characteristic (ROC) curves illustrate the diagnostic accuracy of the salivary metabolites in distinguishing AMI patients from healthy controls. The Area Under the Curve (AUC) values indicate the overall performance, with higher AUC reflecting better discriminatory ability. Statistical significance is denoted by *p* < 0.05. SH represents the healthy group, and SP represents the AMI group.

To further assess the potential of the salivary metabolomic profile as a diagnostic tool for AMI, we performed receiver operating characteristic (ROC) analysis to evaluate the accuracy of salivary metabolites in distinguishing AMI patients from healthy controls. Twenty metabolite biomarkers, including Allyl Formate, Altretamine, C75, 4-Hydroxy-2-Methyl-3-Oxo-4-[(2E,6E)-Farnesyl]-3,4-Dihydroquinoline 1-Oxide, Hexylbenzene, 20-HETE Ethanolamide, Cadalene, 6-[51-Ladderane-1-Hexanol, 9(R)-HODE, Nopol, Tanacetol B, 3-Methyl-5-Propyl-2-Furannonanoic Acid, Vetiveryl Acetate, 5-(N-Methyl-4,5-dihydro-1H-pyrrol-2-yl) Pyridine-2-ol, and Tetrahydrobiopterin metabolites were upregulated, and 9-Hydroxy Octadecanoic Acid, PA(19:0/20:0), Hexylamine, Norisodomesticine, and 8-Hydroxycarvedilol were downregulated with an area under curve (AUC) of 0.82 – 0.88 (**Figure 6**), providing strong discriminatory power between AMI and control groups. However, the high diagnostic accuracy requires validation in larger, independent cohorts to confirm its generalizability and mitigate overfitting concerns.

### Alterations in functional pathways of salivary metabolites in AMI

**Figure 7:**
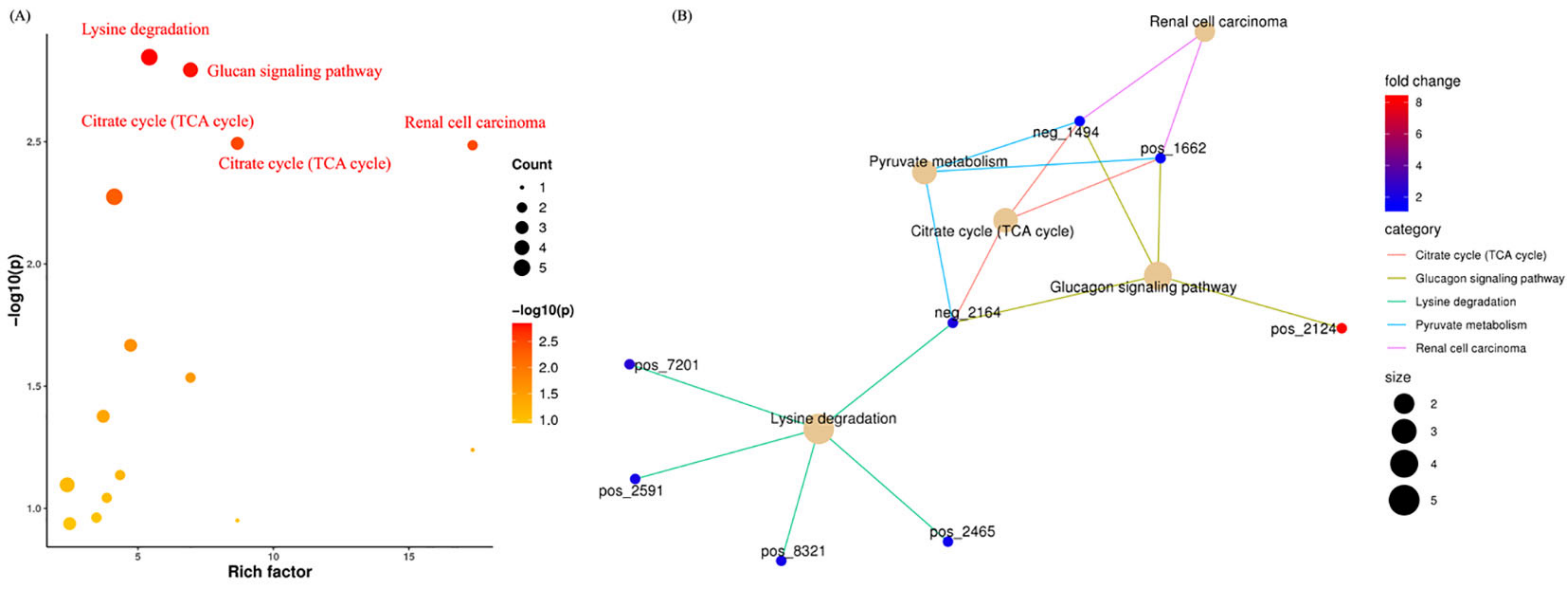
Metabolomics functions, pathways, and their associated metabolites in AMI. **(A)** Indicates the significant enrichment pathways associated with AMI. **(B)** Shows the significant pathways and their corresponding metabolites.

PICRUSt2 was used to predict KEGG pathways, facilitating comparative metabolomic profiling between control and AMI groups. Our study identified five major metabolic pathways that were significantly upregulated, including the Citrate cycle (TCA cycle), Glucagon signaling, Lysine degradation, Pyruvate metabolism, and Renal cell carcinoma (**Figure 7A**). These findings suggest that AMI is associated with significant metabolic disruptions, including altered energy production (TCA cycle, pyruvate metabolism), amino acid breakdown (lysine degradation), and glucose regulation (glucagon signaling), reflecting systemic metabolic dysregulation. Additionally, the involvement of the renal carcinoma pathway indicates potential overlap with broader disease mechanisms.

**Figure 7B** shows the contribution of distinct metabolites in these predicted pathways. For instance, in *Renal Cell Carcinoma*, malic acid and L-malic acid were found to be downregulated. In the *Pyruvate Metabolism* and *Citrate Cycle*, succinic acid, malic acid, and L-malic acid were downregulated. In the *Glucagon Signaling Pathway*: D-glucose 1-phosphate was upregulated, and Succinic acid, L-malic acid, and Malic acid were downregulated. Moreover, in the *Lysine degradation pathway*, (3S,5S)-3,5-Diaminohexanoate, 5-Aminopentanal, D-Lysopine, Succinic acid, and Pipecolic acid were significantly downregulated. The OTUs referenced in the “**Figure 7B**” network plot and their corresponding metabolites are detailed in **Table 1**.

**Table 1:** The metabolites are implicated in distinct pathways closely associated with the pathophysiology of AMI.

Renal cell carcinoma	Pyruvate metabolism and citrate cycle (TCA cycle)	Glucagon signaling pathway	Lysine degradation
*neg_1494*: Malic acid	*neg_1494*: Malic acid	*pos_2124*: D-Glucose 1-phosphate	*neg_2164*: Succinic acid
*pos_1662*: L-Malic acid	*pos_1662*: L-Malic acid	*pos_1662*: L-Malic acid	*pos_2465:* Pipecolic acid
	*neg_2164*: Succinic acid	*neg_1494*: Malic acid	*pos_8321:* D-Lysopine
		*neg_2164*: Succinic acid	*pos_2591:* 5-Aminopentanal
			*pos_7201:* (3S,5S)-3,5-Diaminohexanoate

### Correlation between clinical markers, microbiome composition, and metabolite profiles

**Figure 8:**
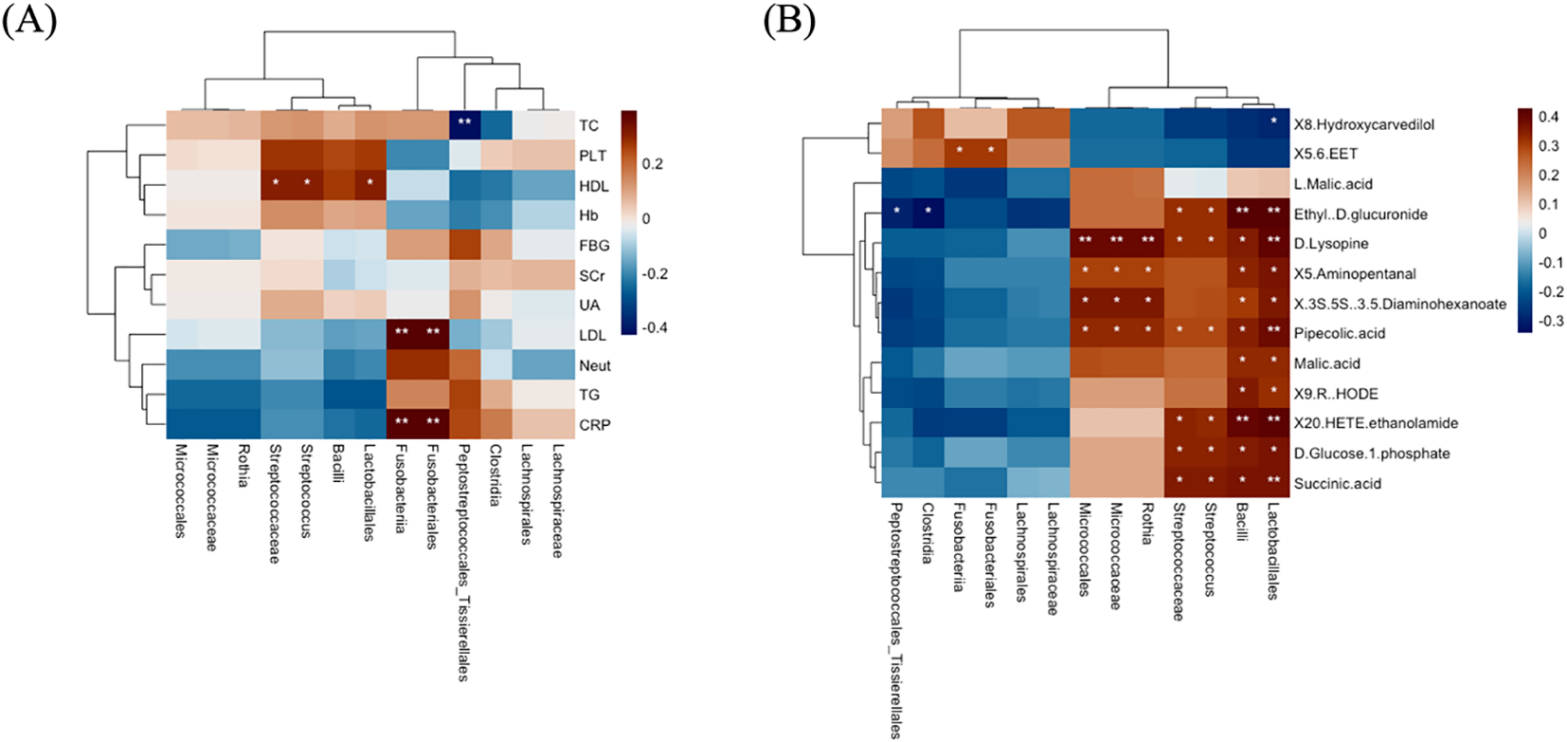
The correlations of distinct oral microbiota with clinical markers and metabolites. **(A)** Oral bacterial associations with clinical markers. **(B)** Oral bacterial correlation with metabolites. In the heatmaps, brown shows positive correlations, blue shows negative, and significance is marked as *P =< 0.05, **P =< 0.01.

Based on the 14 discriminative taxa found by LEfSe, we next tested for their correlations with clinical indices and metabolites using Spearman’s correlation. Our findings showed that AMI-depleted Peptostreptococcales_Tissierellales was negatively correlated with total cholesterol, indicating that this oral taxon could have a beneficial effect on lipid metabolism, possibly reducing cholesterol levels. In addition, AMI-depleted Fusobacteriales and Fusobacteriia were positively correlated with C-reactive protein and LDL-C. The contrasting presence of Fusobacteriales and Fusobacteriia in AMI patients and controls suggests that these taxa might serve as potential biomarkers for cardiovascular health. Furthermore, Lactobacillales, *Streptococcus*, and Streptococcaceae were positively correlated with HDL-C (**Figure 8A**); a higher presence of these taxa could favorably influence lipid profiles, potentially reducing cardiovascular risk by boosting HDL-C levels. Lactobacillales, *Streptococcus*, and Streptococcaceae may play a dual function in myocardial progression. While they are associated with HDL-C and beneficial effects on lipid metabolism and inflammation, their roles in microbial dysbiosis may also contribute to altered metabolic pathways and systemic inflammation, potentially exacerbating cardiovascular risk factors. This dual function highlights the complex interplay between oral microbiota and myocardial health, necessitating further research to clarify their exact contributions.

Additionally, the correlation between distinct microbiota and metabolites found in different pathways was analyzed. We found that the taxa identified in AMI patients were associated with almost all metabolites, while the distinct association with metabolites was rare. For example, AMI-enriched Lactobacillales and Bacilli were positively correlated with Succinic acid, D-Glucose 1-phosphate, 20-HETE ethanolamide, 9(R)-HODE, Malic acid, Pipecolic acid, (3S,5S)-3,5-Diaminohexanoate, 5-Aminopentanal, D-Lysopine, and Ethyl-D-glucuronide. In addition, MI-enriched *Streptococcus* and Streptococcaceae were positively correlated with Succinic acid, D-glucose 1-phosphate, 20-HETE ethanolamide, Pipecolic acid, D-lysopine, and ethyl-D-glucuronide. Furthermore, AMI-enriched *Rothia*, Micrococcaceae, and Micrococcales were positively correlated with Pipecolic acid, (3S,5S)-3,5-Diaminohexanoate, 5-Aminopentanal, and D-Lysopine. Meanwhile, AMI-depleted Fusobacteriales and Fusobacteriia were positively associated with 5,6-EET metabolite, while Clostridia and Peptostreptococcales_Tissierellales were negatively correlated with Ethyl-D-glucuronide (**Figure 8B**). The findings indicate that AMI-enriched taxa (e.g., Lactobacillales and *Streptococcus*) are broadly associated with metabolites linked to energy, carbohydrate, and amino acid metabolism, potentially reflecting metabolic shifts in AMI patients. In contrast, AMI-depleted Fusobacteriales and Fusobacteriia, associated with anti-inflammatory metabolites like 5,6-EET, suggest their loss could exacerbate inflammation and cardiovascular risk. These insights highlight the microbiome’s significant role in modulating metabolic and immune pathways in AMI.

## Discussion

Recently, the impact of microbiota on CVDs has been receiving more and more attention (Jin et al., 2021). Studies have reported that oral and gut microbiota contribute to the development of MI (Kwun et al., 2020). The association between the oral microbiome and metabolome in AMI patients and healthy controls remains largely unexplored. Herein, we investigated the oral microbiome and metabolome in AMI patients and healthy controls using 16S rRNA gene sequencing and LC-MS-based metabolomics. We discovered significant differences in terms of microbial composition, diversity, and specific metabolite biomarkers between AMI and control subjects. These significant findings not only help identify disease-related markers but also provide valuable insights into the underlying mechanisms of AMI.

Our study has strengthened the link between oral microbiome/metabolome and AMI. We found that the oral genera, particularly *Streptococcus* and *Rothia*, were significantly altered in AMI patients, consistent with the previous studies (Kwun et al., 2020; Li et al., 2024). One study found higher levels of *Streptococcus* and *Rothia* in atherosclerotic patients (Kato-Kogoe et al., 2022). Another study found that the genera *Streptococcus*, *Rothia*, and *Corynebacterium* were detected in both the oral cavity and the atherosclerotic plaques of the same patients who underwent carotid endarterectomy for minor ischemic stroke or transient ischemic attack (Koren et al., 2011). A previous study demonstrated higher levels of *Streptococcus* in the atherosclerotic plaques, and its levels were elevated in the oral cavities of patients with atherosclerosis (Ji et al., 2021; Kato-Kogoe et al., 2022). *Streptococcus* isolated from atherosclerotic plaques suggests that the microbiota in these plaques may originate from the oral cavity or gut (Menon et al., 2017, 2020). Long ago, it was realized that *Streptococcus* residing in the oral cavity could eventually gain access to the bloodstream and cause infective endocarditis (Murray et al., 2024). *Streptococcus* receptor polysaccharides may stimulate aortic endothelial cells, and cytokines (IL-6, IL-8, and monocyte chemoattractant protein-1) and intercellular adhesion molecule-1 showed increased expression, thus contributing to cardiovascular disease progression and arterial thrombosis (de Toledo et al., 2012). Therefore, our study corroborated the possibility of these bacterial genera being related to AMI.

Our study also identified 9(R)-HODE, 20-HETE ethanolamide, 8-Hydroxycavedilol, and 5,6 EET as biomarkers for the progression of AMI. 9-HODE, an oxylipin, is an oxidized metabolite of linoleic acid, the most abundant polyunsaturated fatty acid in human diets (Nieman et al., 2014). Several studies have demonstrated the contribution of oxylipins, in general, to cardiovascular diseases (Nayeem, 2018). A study suggested that 9-HODE is associated with pulmonary arterial hypertension (Petriello et al., 2018). 9-HODE was considered among the most important derivatives after an early incident of ischemic stroke (Szczuko et al., 2020). Furthermore, 9-HODE can affect vascular cells, including smooth muscle cells (Szczuko et al., 2020). Alterations in vascular smooth muscle cell signaling and function can affect vascular reactivity and tone, critical determinants of vascular resistance and blood pressure (Touyz et al., 2018). Evidence showed that HODE can induce pro-inflammatory effects, including the production of inflammatory cytokines IL-1β and IL-8 (Ku et al., 1992; Terkeltaub et al., 1994) and the activation of NF-κB (Ogawa et al., 2011). In addition, 9-HODE has been considered a biomarker for oxidative stress and is linked to various pathological conditions such as atherosclerosis, diabetes, chronic inflammation, obesity, and cancer (Nieman et al., 2014, 2016). As oxidative stress plays an important role in the progression of hypertension, there is a strong relationship between 9-HODE and oxidative stress (Rodrigo et al., 2011). The precise role of 9-HODE in the pathophysiology of AMI remains incompletely understood. Advanced investigations are warranted to delineate its underlying mechanisms and assess the therapeutic potential of targeting 9(R)-HODE in the management of AMI. In addition, 20-HETE ethanolamide, a derivative of 20-HETE, plays a complex role in MI by potentially balancing detrimental and protective effects (Zhang et al., 2020). While 20-HETE is known for its pro-inflammatory and vasoconstrictive actions that can exacerbate ischemic injury, the ethanolamide form may modulate these effects differently (Waldman et al., 2016). In the context of AMI, 20-HETE ethanolamide could contribute to vascular dysfunction and inflammation, worsening myocardial damage, but it may also exert cardioprotective effects by attenuating excessive inflammatory responses or oxidative stress (ElKhatib et al., 2023). Its precise role remains unclear, highlighting the need for further investigation into its impact and therapeutic potential in AMI.

Furthermore, 8-Hydroxycarvedilol, a key metabolite of carvedilol, exhibits potent antioxidant and anti-inflammatory properties that protect myocardial cells by mitigating oxidative stress and inflammation critical in post-infarction remodeling (Beţiu et al., 2022). Its downregulation in MI patients, likely due to altered metabolism, may reduce these cardioprotective effects, underscoring the need to optimize carvedilol therapy or enhance metabolite activity in such patients. Moreover, 5,6-EET, a cardioprotective lipid mediator, promotes vasodilation, reduces inflammation, and prevents cardiomyocyte apoptosis, which is crucial for myocardial protection (Isse et al., 2022; Zhang et al., 2022; ElKhatib et al., 2023; Lan et al., 2024). Its downregulation in AMI may worsen ischemic injury by increasing oxidative stress and inflammation, suggesting therapeutic potential for strategies that preserve or enhance 5,6-EET activity. Further mechanistic studies are needed to elucidate the precise roles of 9(R)-HODE, 20-HETE ethanolamide, 8-Hydroxycarvedilol, and 5,6-EET metabolites in the pathophysiology of AMI. This will aid in understanding their contributions to vascular dysfunction, oxidative stress, and inflammation, and explore their potential as therapeutic targets for improving cardiovascular outcomes.

Moreover, our findings demonstrated the dysregulations of Glucagon signaling, TCA cycle, Pyruvate metabolism, Renal cell carcinoma, and Lysine degradation pathways in AMI. The metabolites involved in these pathways were malic acid, L-malic acid, succinic acid, D-glucose 1-phosphate, Pipecolic acid, D-Lysopine, 5-Aminopentanal, and 3,5-Diaminohexanoate. The involvement of metabolites such as malic acid, L-malic acid, succinic acid, and D-glucose 1-phosphate in pathways like Glucagon signaling, the TCA cycle, Pyruvate metabolism, and Renal cell carcinoma pathways in AMI patients suggests significant metabolic reprogramming during MI (Hoang et al., 2021). These pathways reflect critical disruptions in energy production, glucose metabolism, and cellular stress responses (Yu et al., 2021; Nguyen and Schulze, 2023; Wang et al., 2024). Altered TCA cycle activity indicates impaired mitochondrial function, while changes in glucagon signaling and pyruvate metabolism suggest adaptations in glucose and energy homeostasis (Icard et al., 2021; Guo et al., 2022). Additionally, the overlap with renal cell carcinoma pathways may highlight shared mechanisms of metabolic dysregulation and oxidative stress (Fan et al., 2024). Collectively, these findings point to a complex interplay between energy metabolism and cellular stress in AMI, emphasizing the need for further studies to explore their diagnostic and therapeutic potential.

Our study also found a significant disruption in the lysine degradation pathway between the two groups. This pathway was linked to pipecolic acid, which is also implicated in various diseases, including diabetic corneal stroma in humans (Priyadarsini et al., 2016). Dysregulation of lysine degradation pathways may contribute to the early onset of cardiac hypertrophy, indicating that related metabolites could serve as predictive markers and potential targets for intervening in subclinical cardiomyocyte hypertrophy (Liu et al., 2021). Therefore, it is essential to further explore the lysine degradation pathway in T2D to identify possible biomarkers and better understand the underlying biological mechanisms (Razquin et al., 2019). Furthermore, (3S,5S)-3,5-Diaminohexanoate, 5-Aminopentanal, and D-Lysopine metabolites were associated with the lysine degradation pathway. Taken together, the results suggest that salivary metabolites could serve as potential risk factors for AMI, with the oral microbiota playing a significant role in their association.

Moreover, significant correlations were observed among oral microbiota, clinical indices, and metabolites, highlighting their interconnected roles in disease pathology. For instance, the correlation between oral microbiota, specifically AMI-enriched *Streptococcus* and Streptococcaceae, and metabolites such as succinic acid, D-glucose 1-phosphate, 20-HETE ethanolamide, pipecolic acid, D-lysopine, and ethyl-D-glucuronide suggests a potential link between oral microbiota dysbiosis and altered metabolic pathways in AMI. These metabolites are involved in key pathways such as glucagon signaling, the TCA cycle, pyruvate metabolism, and renal cell carcinoma pathways, indicating disruptions in energy metabolism, glucose regulation, and oxidative stress. The association with these pathways highlights the role of oral microbiota in influencing systemic metabolic processes and their potential contribution to the pathophysiology of AMI. Further research is needed to elucidate the mechanistic interactions between oral microbiota and metabolic dysregulation in cardiovascular diseases. Similarly, AMI-enriched Lactobacillales and Bacilli were positively correlated with metabolites such as succinic acid, D-glucose 1-phosphate, 20-HETE ethanolamide, 9(R)-HODE, malic acid, pipecolic acid, (3S,5S)-3,5-diaminohexanoate, 5-aminopentanal, D-lysopine, and ethyl-D-glucuronide. These metabolites are involved in key metabolic pathways, including glucagon signaling, the TCA cycle, pyruvate metabolism, and renal cell carcinoma pathways in AMI patients. The association suggests a potential role of Lactobacillales and Bacilli in influencing metabolic dysregulation, oxidative stress, and inflammatory processes, contributing to the pathophysiology of myocardial infarction. Further studies are needed to clarify their mechanistic involvement and therapeutic potential in AMI.

### Limitations and future research directions

Although this research provides valuable insights, there is still room for future exploration in the study: (i) A key limitation of this study is the small sample size, which may reduce the ability to detect subtle differences in microbiome and metabolomic profiles, increasing the likelihood of false negatives. To further validate our findings, a large-scale randomized controlled trial is necessary. (ii) The cross-sectional design precludes determining whether the observed changes in microbial taxa and metabolites are causative in AMI pathogenesis or a secondary consequence of disease processes. (iii) Lifestyle factors such as smoking or exercise may shape microbial composition, and dietary habits could influence metabolite profiles while introducing potential biases in the observed associations.

Future studies should focus on the following key areas: (i) Longitudinal studies tracking microbiome and metabolomic dynamics from pre-MI to post-MI recovery to identify predictive and prognostic markers. (ii) Integration of metagenomics, metabolomics, and transcriptomics to elucidate microbial functional pathways and their metabolic outputs, with multicenter collaborations across geographically and ethnically diverse populations to enhance statistical power and address inter-individual variability. (iii) Investigation of the roles of key metabolites (e.g., 9(R)-HODE, 8-Hydroxycavedilol, 5,6 EET, and 20-HETE ethanolamide) in AMI pathophysiology using animal models or *in vitro* assays. (iv) Evaluation of the therapeutic potential of targeting the TCA cycle, Pyruvate metabolism, and Glucagon signaling pathways in preclinical MI models to mitigate myocardial infarction pathophysiology effectively. (v) Validation of identified biomarkers in independent cohorts to assess their reliability and utility for early AMI detection.

## Conclusions

This study represents a pioneering effort in integrating microbiome and metabolome profiling within the context of AMI, establishing a microbiological framework for identifying novel biomarkers and therapeutic targets in cardiovascular pathology. Specifically, the detection of AMI-enriched genera, including *Streptococcus* and *Rothia*, and 9(R)-HODE, 20-HETE ethanolamide, 8-Hydroxycavedilol, and 5,6 EET metabolites, highlights their potential as key indicators of AMI progression. These microbiome and metabolomic signatures are promising as non-invasive diagnostic tools, facilitating early detection and risk stratification in clinical settings. The findings illuminate the critical role of these microbial taxa in the pathophysiological mechanisms underlying AMI, thereby emphasizing their relevance in the development of future therapeutic interventions. Moreover, this study contributes to the broader understanding of cardiovascular research, enriching the emerging field of microbiome-metabolome interactions, which holds substantial implications for immunological processes, metabolic regulation, and microbiological sciences. Future research endeavors should prioritize longitudinal investigations to elucidate causal relationships, adopt multiomics strategies to integrate functional genomics with metabolomics, and conduct validation studies in larger, more diverse cohorts to enhance generalizability and clinical applicability.

## Data Availability

The datasets utilized in this study have been submitted to the National Center for Biotechnology Information (NCBI) under the project accession number PRJNA1174709.
